# Neuroinflammation as a Novel Therapeutic Frontier for Sanfilippo Syndrome

**DOI:** 10.3390/children12111530

**Published:** 2025-11-12

**Authors:** Donato Rigante, Chiara Veredice

**Affiliations:** 1 Department of Life Sciences and Public Health, Fondazione Policlinico Universitario A. Gemelli IRCCS, 00168 Rome, Italy; 2 Università Cattolica Sacro Cuore, 00168 Rome, Italy; 3 Pediatric Neurology Unit, Department of Woman and Child Health and Public Health, Fondazione Policlinico Universitario A. Gemelli IRCCS, 00168 Rome, Italy; chiara.veredice@policlinicogemelli.it

**Keywords:** lysosomal storage disorders, mucopolysaccharidosis, glycosaminoglycans, neuroinflammation, interleukin-1, inflammasome, innate immunity, anakinra

## Abstract

Glycosaminoglycans (GAGs), also named ‘mucopolysaccharides’, are nodal constituents of the connective tissue matrix which go through synthesis, demolition, and reconstruction within several cellular structures: an abnormal GAG catabolism is the basis of progressive intra-lysosomal accumulation of non-metabolized GAGs, defining *all* mucopolysaccharidoses (MPS), protean disorders characterized by physical abnormalities and multi-organ failure depending on the specific site of non-renewable GAGs stored. A severe cognitive decline is typically observed in the Sanfilippo syndrome, which corresponds to MPS type III, a group of four inherited neurodegenerative diseases resulting from the lack of specific enzymes involved in heparan sulfate (HS) metabolism. As a consequence, the storage of partially degraded HS fragments within lysosomes of the central nervous system elicits chain inflammatory reactions involving the NLRP3-inflammasome in microglia and astrocytes, which cease their homeostatic and immune functions and finally compromise neuron survival. This article provides an overview of the neuroinflammatory picture observed in children with MPS type III, postulating a role of HS accumulation to prime innate immunity responses which culminate with pro-inflammatory cytokine release in the brain and highlighting the relevance of interleukin-1 as a main contributor to neuroinflammation.

## 1. Introductory Cues About the Mucopolysaccharidoses

The mucopolysaccharidoses (MPS) are a group of lysosomal storage disorders caused by the inability to degrade glycosaminoglycans (GAGs), long-chain negatively charged linear sugars with repeating disaccharide units, also named ‘mucopolysaccharides’, which may be related to the absence or partially deficient activity of specific lysosomal enzymes: the resulting clinical manifestations affect various organ and body systems, depending on the type of enzyme involved and peculiarity of non-metabolized GAGs [1]. These molecules are normally found in proteoglycan structures, and their lysosomal degradation is a stepwise mechanism where individual sugars are removed from the end of molecules through a domino-organized enzyme pathway, which is different according to the different GAGs: heparan sulfate (HS), dermatan sulfate, keratan sulfate, chondroitin sulfate, and hyaluronic acid. Eleven primary GAG-related metabolic disorders have been identified, with various scenarios originating from an inadequate breakdown of GAGs, which accumulate in the cell lysosomes of affected individuals, leading to lysosome rupture and extracellular release of abnormally degraded GAGs and other substances. This framework generates complex clinical symptoms starting during childhood, although onset and severity may be quite variable, which include stunted growth, dysostosis multiplex originating from flawed endochondral and membranous ossification, vision and hearing impairment, heart valve disease, organomegaly, and many others [2,3,4]. Psychomotor retardation is often present in MPS, and—if combined with face similarity to gargoyles, coarse visage, macrocrania, and grotesque features—should always arouse the suspicion of MPS [5]. Central nervous system abnormalities can be heralded by impaired skills of cognition, hyperactive and/or aggressive behaviors, epilepsy, hydrocephalus, and severe sleeping problems throughout infancy, correlated with altered specific GAG metabolism within central nervous system cells; these phenomena are frequently observed in different MPS types: I (in its severe and intermediate form), II, III, and VII. An overall classification of the MPS has been reproduced in Table 1. From the therapeutic viewpoint, the currently approved treatment in MPS types I, II, IV-A, VI, and VII is enzyme replacement therapy (ERT), which has been proven to ameliorate walking ability, pulmonary function, and long-term endurance of such patients, as demonstrated by many data from trials and longitudinal studies. However, cardiac valve disease, either joint or skeletal disease, and any pre-existing neurocognitive impairment do not generally improve after ERT if pathological changes had already taken place [6]. In addition, hematopoietic stem cell transplantation is fully indicated for the MPS I at an early stage, with donor-derived cells being able to traffic across the blood–brain barrier and cross-correct cells in the brain [7].

## 2. The Puzzle of Sanfilippo Syndrome

**Figure 1:**
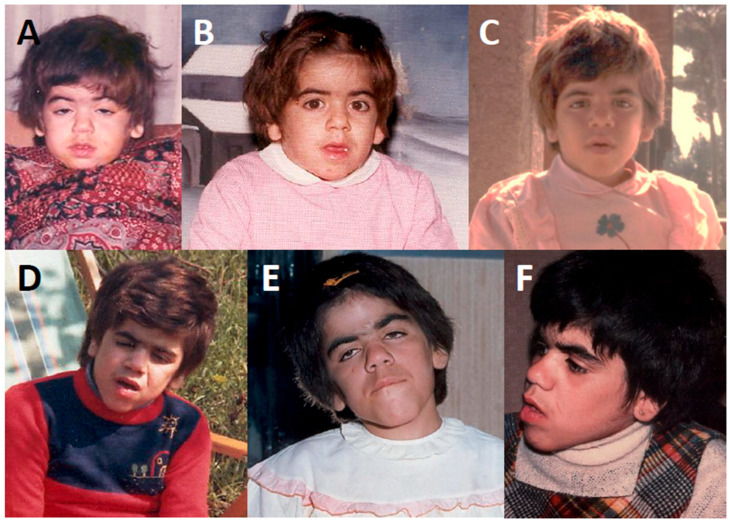
A female-child diagnosed with Sanfilippo syndrome in pictures shot at the age of 2 (**A**), 3 (**B**), 5 (**C**), 6 (**D**), 8 (**E**), and 10 years (**F**); her parents asked for many clinical consultations due to a mix of impoverished social interaction, severe abolition of sleep, epilepsy, and psychomotor regression starting at around 4 years. The diagnosis of mucopolysaccharidosis type III-A was established at 7 years via skin biopsy and specific enzyme activity measurement on peripheral blood leukocytes. Both parents of this child have given their written permission to publish the picture of their daughter.

One of the most enigmatic MPS occurring in childhood is Sanfilippo syndrome, an autosomal recessively transmitted condition caused by the deficiency of four different lysosomal enzymes leading to progressively severe central nervous system degeneration with the decline of both social and adaptive skills: it is also known as MPS type III and is divided into four subtypes (named A, B, C, and D) according to the specific deficiency of the enzymes *N*-sulfoglucosamine sulfohydrolase, alpha-*N*-acetylglucosaminidase (or NAGLU), alpha-glucosaminide *N*-acetyltransferase, and *N*-acetylglucosamine-6-sulfate sulfatase, respectively, all acting at different levels in HS catabolism [8]. Undegraded HS accumulates within lysosomes of brain (and other) cells, disrupting their function and unaccountably eliciting neurologic abnormalities, psychomotor impairment, and most importantly also disarming features of the autism spectrum disorder: Sanfilippo syndrome’s clinical course can be devastating with gradual deterioration in speech and mobility and also decreased lifespan; in fact, it is an influential cause of dementia in young patients after an essentially normal neurodevelopment during early childhood [9]. Figure 1 depicts the changing features of face over time in a child with Sanfilippo syndrome.

Despite the availability of ERT and many attempts of substrate reduction therapies for some types of MPS, at present, there are no approved treatments for children with Sanfilippo syndrome, with death commonly occurring in the second decade [10]. A newborn screening for this syndrome would probably enable pre-symptomatic diagnosis and optimize any potential therapeutic benefit, though current opportunities for patients with MPS type III are limited to palliative care. Recent research focused on gene therapy and pharmacological chaperones to target the improperly folded enzyme as well as substrate reduction therapy with genistein (4′,5,7-trihydroxyisoflavone), a specific inhibitor of tyrosine kinase, resulting in the attenuation of astrogliosis within the cerebral cortex of mice with MPS type III-B [11]. A one-year pilot study showed that genistein-rich soy isoflavone improved hair morphology and cognitive functions in pediatric patients with MPS III-A and III-B [12]. However, a more recent study did not support the use of genistein aglycone in MPS type III, as no clinically meaningful improvements in neuropsychological outcomes were found [13].

Pre-clinical studies have also shown that intracerebro-ventricular ERT with a fusion protein of the recombinant human NAGLU combined with insulin-like growth factor 2 (named alpha-tralesinidase) in a mouse model of MPS type III-B increased enzyme activity in brain homogenate even after a single injection given at birth [14]. Accordingly, a second phase I/II open-label study related to 22 patients with MPS type III-B, all treated with intracerebro-ventricular alpha-tralesinidase at the mean age of 60 months, revealed normalization of both liver and spleen sizes, and also improvement of the developmental quotients in most cases [15].

## 3. The Development of Neurodysfunction in Mucopolysaccharidoses

The relationship between abnormal GAG storage and neurodysfunction in MPS is only partially understood. There is evidence suggesting that the gradual accumulation of GAGs might trigger an immune response culminating with inflammation parallel to the accumulation of secondary immunostimulatory substrates, which also favor neurodegeneration [16]. GAG accumulation in MPS patients’ cells disrupts lysosomal integrity and permeability, affecting autophagic properties and leading to organelle swelling and vacuolation even in neural stem cells [17]. Woodbury & Ikezu proved an HS-mediated abnormal activity of fibroblast growth factor-2, which regulates neural stem cell propagation, proliferation and differentiation and drives dopaminergic neuron and nigro–striatal pathway development in vivo, resulting in altered synapse plasticity and axonal branching within the subventricular and subgranular zones of the hippocampal dentate gyrus in animal models with neurodegenerative disorders [18]. Increased oxidative stress caused by perturbed mitochondrial activity has been described in many types of MPS, which could contribute to worsen inflammation through the release of reactive oxygen species (ROS) and reactive nitrogen species, also influencing lysosome homeostasis [19]. Indeed, mice models of different MPS types have shown a progressive neuronal loss within the cerebellum and increased levels of gangliosides and ROS within the brain, having detrimental effects on glial cell function [20]. Pathways connected with inflammation and oxidative stress have also been highlighted in other lysosomal storage diseases with neurologic involvement as components of their neuropathology; in particular, the absence of enzymes required for HS degradation leads to the formation of aberrant fragments of GAG chains, which have been found in the plasma and urine of Sanfilippo patients, having the potential to interact with Toll-like receptors (TLR), specifically TLR4, involved in the production of pro-inflammatory cytokines, key-mediators of the innate immune response by cells that require a fine regulation to maintain a balance between pro- and anti-inflammatory activity: this balance is critical for regulating synapse formation, cell renewal processes, and apoptosis of cells within the central nervous system.

Disrupted cytokine and chemokine expression during early neurodevelopmental periods substantially influence microglia, astrocytes, and neural progenitors [21]. Maccari et al. found an increased level of sulfation in the HS of brain, particularly predominant in position 2 of uronic acid, in a mouse model of MPS type III-A, demonstrating for the first time a common feature to visceral and nervous tissues caused by the lack of physiological sulfamidase activity and supposing its potential involvement in both systemic disease progression and neuropathology [22]. Accumulation of excessive abnormally sulfated HS has the potential to influence many downstream cellular functions: Wilkinson et al. analyzed mouse brains of MPS type I, type III-A and III-B at 4 and 9 months of age in comparison with wild-type mouse brains via quantitative immunohistochemistry and transmission electron microscopy to establish the events that caused neurodegeneration and provide additional outcome measures for MPS treatment strategies. In particular, they found an increased lysosomal storage burden within the cells of the cerebral cortex of MPS brains if compared to wild-type, and most lysosomes also exhibited ganglioside storage with dystrophic axons and reduced myelination occurring at 4 months of age. Analysis of the disaccharide composition of HS from MPS brains indicated that HS in all MPS types was abnormally highly sulfated with a trend for increased sulfation from MPS type I to III-A, with III-B the highest. It has been proposed that excess GAGs in the lysosome caused secondary storage by inhibiting ganglioside-degrading enzymes, which led to decreased or mislocalized synaptic vesicles and decreased post-synaptic proteins, contributing to neuroinflammation, astrocytosis, and microgliosis. Indeed, the authors found a number of pro-inflammatory cytokines, including monocyte chemotactic protein-1, macrophage inflammatory protein-1α (MIP-1α), and interleukin (IL)-1α in the sites of inflammation of MPS-related brain extracts assessed at 9 months compared to wild-type [23].

## 4. Innate Immunity Derangement in Neurologic Disorders

Sentinel cells of the innate immune system including neutrophils, monocytes and their tissue counterparts which are the macrophages become activated through pattern recognition receptors such as TLRs and launch a cell-mediated response via NLRP3-inflammasomes that initiate a pro-inflammatory cascade with final production of IL-1β, IL-18, tumor necrosis factor (TNF)-α, and IL-6, also influencing downstream adaptive responses in some cases. The role of NLRP3-inflammasomes has been disclosed by many studies, suggesting that neuroinflammation is a relevant pathogenetic mechanism in complex neurological diseases such as autism spectrum disorder (ASD). Hughes et al. have shown higher numbers of circulating innate immunity monocytes in children with ASD and higher levels of pro-inflammatory cytokines following upregulated expression of inflammatory genes in tissue-resident immune cells, such as microglial cells and astrocytes, with potential breakage of the brain barrier [24]. Significant elevations of IL-1β, IL-6, and IL-12p40 have been seen in a large cohort of ASD children aged 2–5, compared to typically developing children; moreover, these elevated pro-inflammatory cytokines were associated with worsening behaviors [25]. Similar increases in IL-1β and IL-12 were also seen in a cohort of high-functioning ASD males, along with increases in the physiologic IL-1 receptor antagonist (IL-1Ra) and T helper cell-associated cytokines IL-5, IL-13, and IL-17 [26].

Furthermore, tuberous sclerosis complex (TSC), a systemic disease caused by mutations either in the genes *TSC1* or *TSC2*, may lead to symptoms basically related to neuroinflammation starting in the microglia and involving the NLRP3-inflammasome, with final overproduction of IL-1β [27]. A transcriptome sequencing dataset has been used to explore the different expression of inflammasome-associated genes in a TSC-knockdown microglia model generated by lentivirus, confirming the pro-inflammatory polarization of microglia [28]. Overproduced IL-1β can elicit astrocyte IL-1 receptors to promote transcription of chemokines and danger signals associated with the nuclear factor kappa-light-chain-enhancer of the activated B-cell (NF-κB) pathway [29]. In addition, IL-1β is involved in a chain of events leading to the production of IL-6, cyclooxygenase 2, and matrix metalloproteinases in TSC astrocytes, exacerbating the neuroinflammation [30].

Acute inflammation in these scenarios is mediated by the innate immune system, involving macrophages, dendritic, natural killer and ectodermal cells, which display specific membrane-bound and cytosolic receptors such as TLRs, C-type lectin receptors, retinoic acid-inducible receptors and nucleotide-binding oligomerization domain-like receptors, allowing us to sense damage-associated molecules which ultimately drive pro-inflammatory cytokine release and an inflammatory gasdermin D-mediated form of lytic programmed cell death, called pyroptosis, resulting from the release of the N-terminal fragment of gasdermin D [31]. The progressive accumulation of abnormally fragmented GAGs has been shown to promote TLR4 signaling, and higher TLR4 expression is parallel to significantly increased NF-κB-associated cytokines [32]. Extracellular matrix components like low molecular weight fragmentation products of hyaluronic acid act as damage-associated molecular patterns, serving as endogenous ligands for the TLR4 complex, and activate immunocompetent cells, resulting in distinct phosphorylation of p38/p42/44 MAP-kinases and NF-κB, finally fueling TLR4-dependent inflammation [33]. Inflammasome-related genes have been found hyper-expressed in neural progenitor cells of patients with gangliosidosis, another lysosomal storage disorder in which insufficient β-galactosidase activity leads to abnormal accumulation of complex gangliosides within the central nervous system [34].

Ausseil et al. tried to examine the causative link between HS accumulation, inflammation, and disease markers in the cortex of mice models with MPS type III-B, finding that HS oligosaccharides could prime microglial activation through TLR4 and MyD88 both in vitro and in the MPS type III-B mouse brain, also revealing that neurodegeneration progression is independent by HS-induced stimulation of innate immunity. The authors found that the incubation of normal mouse microglia cultures with MPS type III-B patient-derived HS oligosaccharides triggered TNF-α release and induced higher mRNA expression of both IL-1β and MIP-1α in microglia and astrocytes [35]. Further investigations are required to specify the structure and pattern of sulfation and acetylation of HS oligosaccharides responsible for priming microglial activation. The role of TLR4 has also been documented in Alzheimer’s disease, where it increases the recognition of fibrillar amyloid-beta (Aβ) [36], while mutations in the *NAGLU* gene (encoding the enzyme *N*-acetylglucosaminidase, deficient in MPS type III-B) have been recognized in Parkinson’s disease, suggesting that allelic *NAGLU* changes may increase patients’ susceptibility to develop Parkinson-like phenotypes due to lysosomal dysfunction [37]. Moreover, a secondary accumulation of amyloidogenic proteins such as fibrillar Aβ, α-synuclein, and hyper-phosphorylated tau, which propagate in a prion-like manner throughout the brain, has been specifically recognized in the central nervous system of mice with MPS type III-A and III-B, leading to a vicious cycle which worsens neuronal degeneration [38].

## 5. A Pro-Inflammatory Milieu in Sanfilippo Syndrome Mimicking the Kaleidoscopic Features of Autoinflammation

Several researchers have shown how the production of both peripheral and central nervous system pro-inflammatory cytokines is increased in MPS, and how significantly higher levels of IL-1β, TNF-α, IL-6, and MIP-1α have been detected in the serum of many patients with neuropathic forms of MPS [39]. In particular, IL-1 has been recognized as a seminal contributor to the development of different neurodegenerative disorders [40]. As confirmation, the severity of MPS type III can be attenuated by decreasing inflammation through corticosteroids. This has been shown by an ex vivo haematopoietic stem cell gene therapy approach: Holley et al. transduced MPS type III-B haematopoietic stem cells with a lentiviral vector driving the expression of NAGLU (*N*-acetylglucosaminidase), which was transplanted into busulfan-myeloablated MPS III-B mice and compared at 8 months of age with mice receiving a wild-type transplant. The authors found increased NAGLU activity in the brain of NAGLU-treated mice (8-fold over the wild-type transplant), which improved behavioral abnormalities; the addition of oral prednisolone improved inflammatory aspects further and reduced pro-inflammatory cytokine expression. Importantly, prednisolone treatment alone in wild-type transplants improved many aspects of mice behaviors despite having little effect on brain neuropathology [41].

**Figure 2:**
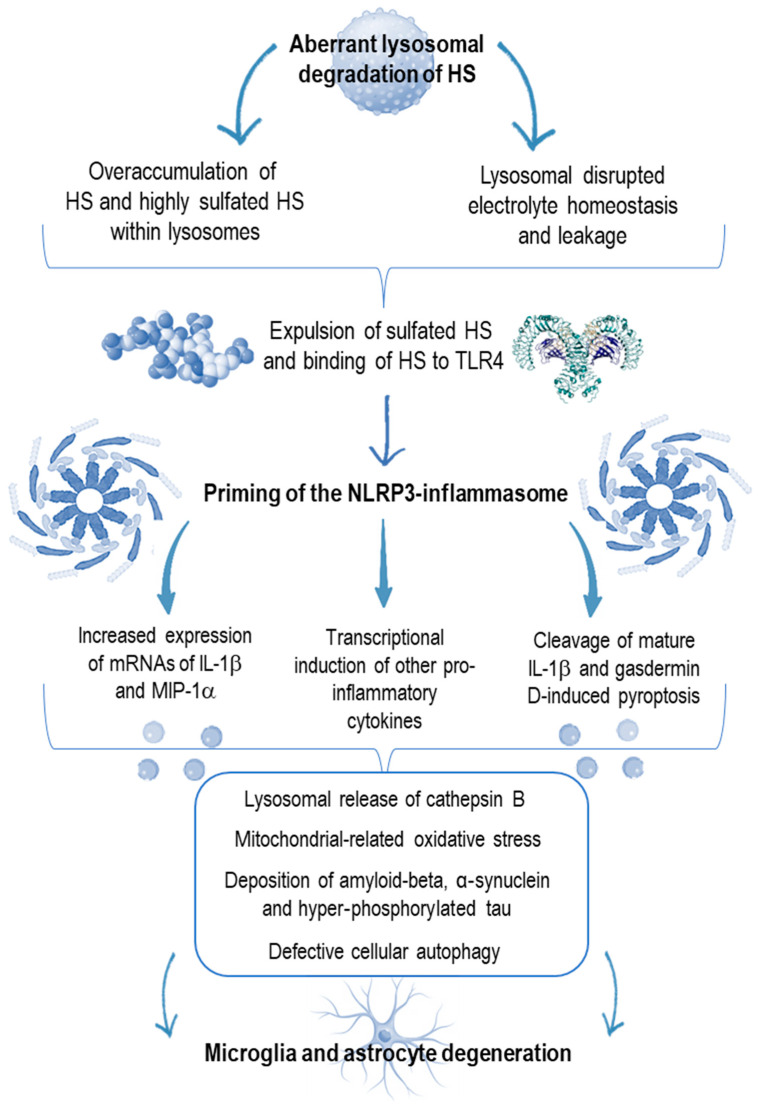
Heparan sulfate/TLR4/interleukin-1 dependent priming of the NLRP3-inflammasome in microglial cells and astrocytes of patients with mucopolysaccharidosis type III, from which interleukin-1-based neuroinflammation erupts. **Notes: HS: heparan sulfate; IL-1β: interleukin-1β; MIP-1α: macrophage inflammatory protein-1α; mRNA: messenger ribonucleic acid; NLRP3: nucleotide-binding oligomerization domain-like receptor family pyrin domain containing 3; TLR4: Toll-like receptor 4.**

In past years, a great scientific advance in genetics and molecular biology has improved our knowledge about genetically based autoinflammatory syndromes which are characterized by seemingly unprovoked IL-1-mediated inflammatory episodes, sometimes concealed behind many cases of recurrent fevers in children [42]. This expanding cluster of diseases includes inflammasome-related disorders like familial Mediterranean fever, TNF receptor-associated periodic syndrome, and mevalonate kinase deficiency [43]. A host of protean manifestations can be depicted in hereditary autoinflammatory diseases, involving the cardiovascular system, serosal membranes, skin, gut or the central nervous system, and different activity scoring systems have been developed for judging both the clinical course and response to treatments [44,45,46]. An alteration of lysosome homeostasis can be sensed by NLRP3-inflammasomes, which cause NLRP3 monomers to oligomerize and interact with the adaptor apoptosis-associated speck-like protein containing a C-terminal caspase recruitment domain (ASC), which recruits pro-caspase-1 and activates caspase-1, fostering both IL-1β and IL-18 maturation and pyroptosis [47,48] (see Figure 2). By using quantitative polymerase chain reaction, Arfi et al. compared the expression of selected genes in cerebral tissues of mice models with MPS type IIIA with non-affected mice, finding several genes over-expressed in the first ones which reflected the inflammation burden as well as apoptosis and oxidative stress severity. The authors also found higher levels of cathepsin B, a cysteine protease playing a role in both NLRP3-inflammasome activation and neuronal death, following an extreme oxidative stress [49]. Moreover, activated microglia might trigger apoptosis in many cellular types of the central nervous system through oxidative burst response and ROS production, which also influences the amyloidogenic processing of amyloid precursor proteins, as seen in Alzheimer’s disease [50]. DiRosario et al. investigated the role of immune responses in the neurological picture of mice with MPS type III-B by means of gene expression microarrays and real-time quantitative reverse transcriptase–polymerase chain reaction, finding significant upregulation of numerous immune-related genes involving a broad range of immune cells and molecules, including T cells, B cells, microglia/macrophages, complement, major histocompatibility complex class I, immunoglobulin, TLRs, and other molecules essential for antigen presentation. The authors also demonstrated for the first time that immunosuppression with prednisolone started at 3–5 weeks of age could significantly slow the central nervous system disease progression and improve behavioral performances, learning ability, and motor functions of an MPS type III-B mouse model [51].

An aberrant activation of the NLRP3-inflammasome has been linked with several inflammatory disorders, which classically include cryopyrin-associated periodic syndrome, but also Alzheimer’s disease, diabetes, atherosclerosis and even autoimmune diseases [52,53,54,55,56]. Inflammasome-based inflammation which starts from sensing cellular stress or infections potentially joins Sanfilippo syndrome with many hereditary autoinflammatory conditions. While all previous data convey the idea that IL-1 steers inflammation in the central nervous system of MPS patients, TNF-α seems otherwise involved in the periphery, mediating both pro-inflammatory and programmed cell death pathways: this cytokine existing as either a transmembrane or a soluble molecule may activate different signaling cascades and downstream genes, and is involved in the regulation of neurogenesis, myelination, blood-brain barrier permeability, and synaptic plasticity. However, it can also potentiate neuronal excitotoxicity and neuroinflammation [57]. Accordingly, TNF-α antagonists have been used to treat various chronic inflammatory conditions, including rheumatoid arthritis, Crohn’s disease, and psoriasis, but also autoinflammatory diseases [58,59,60]. As higher TNF-α production has been reported in animal models of MPS and in patients with MPS types I, II, and III, many efforts have been made with TNF-α inhibitors like infliximab and adalimumab to address the non-neurological manifestations [61]. A further therapeutic target could be the TLR4-TNF-α pathway, and Simonaro et al. confirmed the role of TLR4 signaling in MPS skeletal disease [62]. A combined protocol using the recombinant human *N*-acetyl-galactosamine-4-sulfatase and the rat-specific anti-TNF-α drug has been used to treat the musculoskeletal symptoms in rats with MPS type VI, leading to improve chondrocyte apoptosis, skeleton morphology, and overall motor activity [63].

## 6. Targeting Neuroinflammation as a New Goal in the Sanfilippo Syndrome

Indirect proof that neuroinflammation is an integral part of the neurologic picture of MPS has been given by the positive effects generated by immunosuppression with prednisolone, which improved morphological and functional changes in microglia or astrocytes and significantly slowed central nervous system disease progression in MPS. Furthermore, Kumar et al. showed that an increased expression of microglial pyroptosis-related proteins, frequently co-localized with misfolded protein aggregates, might suggest a platform for the progression of different neurodegenerative disorders along with epigenetic and post-translational modifications of histone and non-histone proteins, which ultimately expedite neurodegeneration [64]. A significant upregulation of many immunoregulatory genes involving a broad range of immune cells has been proved in the brain of mice with MPS III-B by gene expression microarrays and real-time quantitative reverse transcriptase–polymerase chain reaction, justifying the opportunity of repurposing anakinra, the recombinant human IL-1Ra—which revealed an outstanding effectiveness in many inflammatory conditions—as a compassionate/off-label drug for Sanfilippo syndrome [65]. At present, targeting IL-1 with IL-1 antagonists represents the logical therapeutic approach for NLRP3-inflammasome-based autoinflammatory diseases and for NLRP3-related diseases like cryopyrin-associated periodic syndrome, directly caused by gain-of-function *NLRP3* variants [66,67]. Anakinra, acting on both IL-1α and IL-1β, has been successfully used to manage the neurologic picture of the most severe patients with cryopyrin-associated periodic syndrome who display a frank IL-1-mediated chronic meningopathy [68].

A gene therapy-based approach has been used to target the IL-1-dependent neurological manifestations in MPS type III-A and investigate its effects on neuroinflammation: Parker et al. attenuated IL-1 signaling in mice with MPS type III-A using lentiviral-mediated haematopoietic stem cell gene therapy to overexpress IL-1Ra, leading to prevent working memory deficits and mitigate brain glial activation; 2 months after transplant a higher expression of IL-1Ra was confirmed in the plasma of transplanted mice, and there was also a significant IL-1Ra expression in the brain when compared to non-transplanted controls [69]. Additionally, Polgreen et al. started a phase II/III clinical trial with anakinra in patients with MPS type III, aiming to test its safety, tolerability and effects on neurobehavioral outcomes and quality-of-life measurements: 23 patients with an age range of 6-to-26 years were enrolled, with 15 requiring an increased dose at week 8 or at week 16; the administrations of anakinra improved both neurobehavioral and functional outcomes, only at the price of negligible injection site-reactions [70].

Even the use of pharmacological inhibitors of NLRP3-inflammasome as the small-molecule MCC950 might control neuroinflammation in MPS, blocking canonical and noncanonical NLRP3 activation in innate immunity cells, preventing NLRP3-induced ASC oligomerization, and decreasing IL-1β and IL-18 release [71]. Another molecule inhibiting NLRP3–NLRP3 interaction and ASC oligomerization could be the anti-allergic drug tranilast, which was shown to inhibit mast cell cytokine release, fibroproliferative airway changes, macrophage infiltration, transformation of epithelial cells, and also NLRP3 activation [72]. Additional immunomodulatory interventions like co-stimulation blockade with cytotoxic T-cell lymphocyte antigen 4-immunoglobulin (abatacept) acting against CD80 and CD86 of antigen-presenting cells or a depletion of lymphocytes and monocytes through a recombinant humanized monoclonal antibody directed against CD52 (alemtuzumab) or the cumulative suppression of B and T lymphocytes followed by immune cell reconstitution operated by specific drugs like cladribine (a nucleoside analogue of deoxyadenosine) are emerging as novel options for lysosomal storage disorders, though experts in the field suggest that these new treatments should be evaluated on an individual basis through ‘personalized’ trials [73,74]. There are different ongoing studies dedicated to patients with a documented diagnosis of Sanfilippo syndrome to assess their disease natural course and progression and to identify potential surrogate endpoints that may be used in future studies combined with standardized clinical, biochemical, neurocognitive, developmental and imaging measures; some of these trials are in progress (see Table 2).

## 7. Conclusive Remarks

The mechanisms underlying the initiation and perpetuation of immune responses within the central nervous system of patients with MPS remain not fully explained and a potential innate immunity signature has yet to be exactly unraveled for the neuropathic forms of MPS. However, an expanding body of evidence suggests that neuroinflammation is a pathogenetic machinery of utmost importance in many neurological disorders, and that TLR4/IL-1 dependent priming with activation of the NLRP3-inflammasome assembly followed by IL-1β/IL-18 maturation and pyroptosis are determinant in eliciting both neurocognitive decline and behavioral abnormalities in the Sanfilippo syndrome [75]. The progressive accumulation of non-metabolized HS and production of highly sulfated HS fragments recognized as danger signals bring about a sequence of inflammatory cascades, initiated by TLR4 activation, characterized by the final oversecretion of pro-inflammatory cytokines within the central nervous system, resulting in microglia and astrocyte dysfunction. Targeting microglial pyroptosis may offer a potential strategy for neurodegenerative diseases, and immunomodulatory drugs like anakinra could become a promising tool in the management of neuropathic MPS as Sanfilippo syndrome with the aim of minimizing the unmet needs of these complex patients.

Further research will aid in our understanding of how the innate immune system can characterize neuroinflammation in MPS type III, ultimately individualizing the therapeutic strategies and quality of life of patients.

## Figures and Tables

**Table 1:** General details of the mucopolysaccharidoses in humans.

MPS Subtype	Eponym	Causative Gene	Deficient Enzyme	Accumulated Glycosaminoglycans	General Clinical Manifestations
I-H	Hurler	*IDUA*	alpha-L-iduronidase	DS, HS	Coarse face, severe intellectual disability, short stature, dysostosis multiplex, organomegaly, corneal clouding, heart valvular abnormalities
I-S	Scheie	*IDUA*	alpha-L-iduronidase	DS, HS	Joint stiffness, moderate skeletal anomalies, corneal clouding, normal neurologic development
I-HS	Hurler-Scheie	*IDUA*	alpha-L-iduronidase	DS, HS	Less severe clinical manifestations in comparison with Hurler disease
II	Hunter	*IDS*	iduronate 2-sulfatase	DS, HS	Coarse face, short stature, skeletal deformities, organomegaly, heart valvular abnormalities, hearing loss
III-A	Sanfilippo	*SGSH*	*N*-sulfoglucosamine sulfohydrolase	HS	Coarse face, severe regression of neurodevelopment, autism-like behaviors
III-B	Sanfilippo	*NAGLU*	alpha-*N*-acetylglucosaminidase	HS	Coarse face, severe regression of neurodevelopment, autism-like behaviors
III-C	Sanfilippo	*HGSNAT*	alpha-glucosaminide *N*-acetyltransferase	HS	Coarse face, severe regression of neurodevelopment, autism-like behaviors
III-D	Sanfilippo	*GNS*	*N*-acetylglucosamine-6-sulfate sulfatase	HS	Coarse face, severe regression of neurodevelopment, autism-like behaviors
IV-A	Morquio A	*GALNS*	*N*-acetylgalactosamine 6-sulfatase	KS, CS	Coarse face, severely short stature, skeletal abnormalities, pectus carinatum, joint hypermobility, corneal clouding, hearing loss, normal neurologic development
IV-B	Morquio B	*GLB1*	beta-galactosidase 1	KS	Coarse face, short stature, skeletal abnormalities, joint hypermobility, corneal clouding, hearing loss, normal neurologic development
VI	Maroteaux-Lamy	*ARSB*	*N*-acetylgalactosamine-4-sulfatase	DS	Coarse face, dysostosis multiplex, organomegaly, corneal clouding, hearing loss, normal neurologic development
VII	Sly	*GUSB*	beta-glucuronidase	DS, HS, CS	Coarse face, short stature, severe intellectual disability
IX	Natowicz	*HYAL1*	hyaluronidase	HA	Soft tissue masses, mildly short stature, joint mobility restriction, susceptibility to develop ear infections

CS = chondroitin sulfate; DS = dermatan sulfate; HA = hyaluronic acid; HS = heparan sulfate; KS = keratan sulfate.

**Table 2:** List of some clinical trials completed or in progress for patients with mucopolysaccharidosis type III (Sanfilippo syndrome).

Study Number	Target	Type of MPS	Sponsor	Site	Status
NCT06567769	Safety, tolerability, efficacy, pharmacokinetics and pharmacodynamics of recombinant human heparan *N*-sulfatase administered via intra-cerebroventricular access device (phase 1 study)	III-A	GC Biopharma Corp	Multi-center	Recruiting
NCT02053064	Long-term follow-up of patients treated by intracerebral SAF-301 gene therapy	III-A	Lysogene	Amsterdam, The Netherlands; Manchester, United Kingdom	Completed (for 4 patients)
NCT02060526	Safety and efficacy of recombinant human heparan-*N*-sulfatase via intrathecal drug delivery in a randomized controlled open-label study (phase 2 study)	III-A	Shire	Multi-center	Completed (for 21 patients)
NCT04360265	Safety, tolerability and efficacy of UX111 (previously known as ABO-102)	III-A	Ultragenyx Pharmaceutical Inc	Multi-center	Enrolling by invitation
NCT01299727	Long-term safety and clinical outcome of recombinant human heparan *N*-sulfatase via intrathecal administration in an open-label extension of the study HGT-SAN-055	III-A	Takeda (Shire)	Amsterdam, The Netherlands; Manchester, United Kingdom	Completed (for 12 patients)
NCT01509768	Disease natural course	III-B	Shire	Multi-center	Completed (for 19 patients)
NCT03300453	Intracerebral administration of adenovirus-associated viral vector containing the human *N*-acetylglucosaminidase cDNA in an open-label study (phase 1–2 study)	III-B	UniQure Biopharma B.V.	Single-center (Paris, Le Kremlin-Bicetre Cedex, France)	Completed (for 4 patients)
NCT05825131	Retrospective and prospective natural history	III-C	Phoenix Nest	Bron, France; Dallas, TX (USA)	Recruiting
NCT05648851	Retrospective and prospective natural history	III-D	Phoenix Nest	Single-center (New York, NY, USA)	Completed (for 10 patients)

## Data Availability

Not applicable.
